# Emergence delirium and postoperative delirium associated with high plasma NfL and GFAP: an observational study

**DOI:** 10.3389/fmed.2023.1107369

**Published:** 2023-07-28

**Authors:** Xingyang Liu, Yanfeng Wang, Jinghan Wu, Chunyan Ye, Daqing Ma, E. Wang

**Affiliations:** ^1^Department of Anesthesiology, Xiangya Hospital, Central South University, Changsha, Hunan, China; ^2^National Clinical Research Center for Geriatric Disorders, Xiangya Hospital, Central South University, Changsha, Hunan, China; ^3^Division of Anaesthetics, Pain Medicine and Intensive Care, Department of Surgery and Cancer, Faculty of Medicine, Imperial College London, Chelsea and Westminster Hospital, London, United Kingdom

**Keywords:** emergence delirium, postoperative delirium, neuronal injury, neuroinflammation, elderly patients

## Abstract

**Background:**

Neuroinflammation and neuronal injury have been reported to be associated with the development of postoperative delirium in both preclinical and clinical settings. This study aimed to investigate the potential correlation between biomarkers of neurofilament light chain and glial fibrillary acidic protein and emergence and postoperative delirium in elderly patients undergoing surgery.

**Methods:**

Patients who developed emergence delirium (*n* = 30) and postoperative delirium (*n* = 32), along with their matched controls, were enrolled after obtaining ethics approval and written informed consent. Delirium was assessed using the Confusion Assessment Method for the Intensive Care Unit or Confusion Assessment Method scale, and blood samples were collected before and after surgery for plasma neurofilament light chain and glial fibrillary acidic protein measurements using a single-molecule array.

**Results:**

The study found that in patients with emergence delirium, the increase in plasma neurofilament light chain protein levels during surgery was significantly higher than in non-delirium patients (*P* = 0.002). Additionally, in patients with postoperative delirium, both the increase in plasma neurofilament light chain protein levels (*P* < 0.001) and the increase in plasma glial fibrillary acidic protein levels during surgery (*P* = 0.008) were significantly higher than in non-delirium patients. Multivariate logistic regression analysis showed that the increase in plasma neurofilament light chain protein was associated with emergence delirium (adjusted OR = 1.872, *P* = 0.005), and the increase in plasma glial fibrillary acidic protein was associated with postoperative delirium (adjusted OR = 1.419, *P* = 0.016). Moreover, the American Society of Anesthesiologists Physical Status Classification and surgical duration were also found to be associated with delirium in elderly patients.

**Conclusion:**

Our findings suggest that emergence delirium is linked to elevated levels of neurofilament light chain, a biomarker of axonal injury, during surgery. Furthermore, in addition to axonal injury, postoperative delirium was also associated with an increase in glial fibrillary acidic protein, a marker of astrocyte activation.

## 1. Introduction

Delirium is a common neurological complication following surgery, especially in the elderly. It is an acute brain dysfunction characterized by fluctuating disturbances of concentration, consciousness, and cognitive function (1–3). The incidence of postoperative delirium ranges from 11% to as high as 51% (4), and it is a risk factor for accelerated age-related cognitive decline (5). Elderly patients who develop postoperative cognitive dysfunction or delirium are three times more likely to experience permanent cognitive impairment or dementia (6, 7). Additionally, patients with postoperative delirium have a higher readmission rate and quadrupled mortality rate than those without delirium (8).

Based on onset time, postoperative delirium can be categorized as emergence delirium (ED) and postoperative delirium (POD) (9, 10). ED occurs during or immediately after emergence from general anesthesia and is observed in all postoperative populations but is more common in children and elderly patients (11). In elderly patients undergoing major surgery, ED independently increases the risk of developing postoperative delirium (9). POD typically appears between postoperative day 1 and 1 week after surgery, with peak incidence occurring on postoperative days 1–3. Delirium symptoms usually resolve within hours to days. The underlying mechanism for different types of delirium is multifactorial and remains unclear (10, 12).

The incidence of delirium is known to increase in elderly patients possibly due to age-related vulnerability in brain function and reduced tolerance to surgery and anesthesia (13–15). Biomarker detection may provide insights into potential brain injuries during surgery. The neurofilament light chain (NfL) is a crucial component of the axonal cytoskeleton that plays a vital role in maintaining neuron structure and function (16, 17). Elevated levels of NfL have been detected in central nervous system disorders linked to axonal injury or degeneration (18). Glial fibrillary acidic protein (GFAP) is the primary intermediate filament in mature astrocytes and is involved in the cytoskeletal development of astrocytes (19). Increased GFAP expression is a hallmark of proliferation in astrocytes, a process highly correlated with brain injury (19, 20). Although some preoperative studies have reported an association between NfL and delirium (21, 22), the relationship between GFAP and delirium remains controversial (23–25). Moreover, there are gaps in the longitudinal analysis of the relationship between NfL, GFAP, and emergence delirium. Further research is necessary to explore the role of changes in NfL and GFAP in delirium and their significance as biological markers of delirium.

The collection of cerebrospinal fluid is an invasive procedure, making it unsuitable for routine clinical practice. However, advances in measurement techniques have shown a strong correlation between NfL and GFAP levels in both cerebrospinal fluid and blood (26). In this study, we measured the concentrations of NfL and GFAP in the preoperative and postoperative plasma of elderly patients in order to investigate the relationship between changes in NfL and GFAP during surgery and the occurrence of emergence delirium and postoperative delirium.

## 2. Methods

### 2.1. Study design

The study was conducted from July 2019 to December 2021 at Xiangya Hospital, Central South University, and approved by the Ethics Committee (201907296). It was registered with the Chinese Clinical Trial Registry (ChiCTR2000031444), and all participants provided written informed consent prior to recruitment. The study data were reported according to the Strengthening the Reporting of Observational Studies in Epidemiology guidelines.

### 2.2. Patients

**Figure 1:**
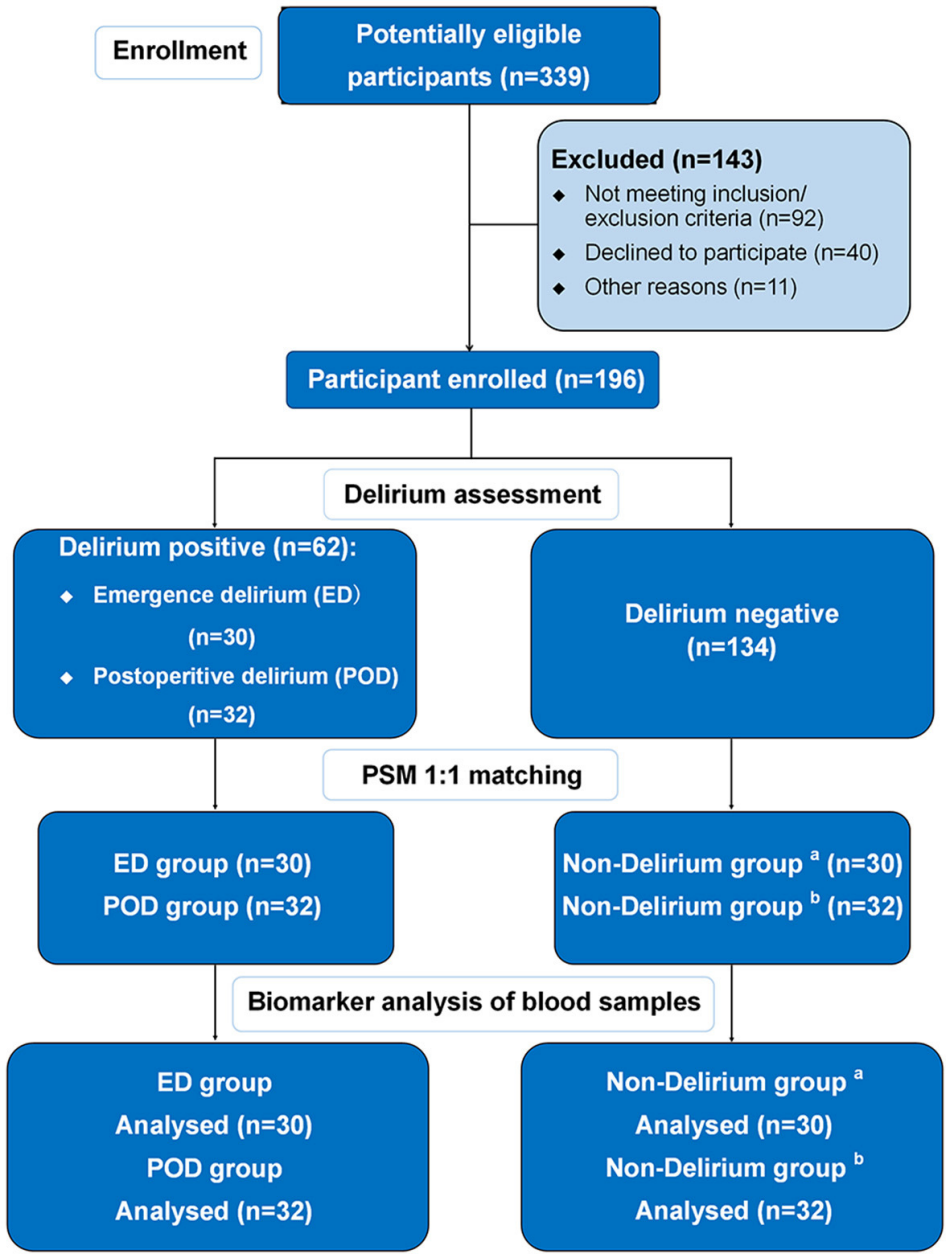
Flow chart. ^a^The control group of the ED group obtained from PSM; ^b^the control group of the POD group obtained from PSM.

The study had the following inclusion criteria: (1) patients aged 60 years or older and (2) elective surgery under general anesthesia. The exclusion criteria were as follows: (1) preoperative neuropsychiatric disorders, such as severe craniocerebral trauma, epilepsy, Alzheimer's disease, other forms of dementia, or schizophrenia; (2) traumatic brain injury or neurosurgery; (3) preoperative diagnosis of dementia; (4) severe hypertension, diabetes, or coronary heart disease; (5) alcohol dependence or withdrawal; (6) participation in other drug or clinical trials within 3 months before study enrollment; (7) inability to perform assessments due to severe visual or hearing impairment; and (8) refusal to participate in the study. Of the 339 elderly patients who underwent surgery, 196 were enrolled in the study. Emergence delirium was assessed using the Confusion Assessment Method for the Intensive Care Unit scale during postoperative care unit stay, while postoperative delirium was evaluated from day 1 after surgery up to postoperative day 7 using the Confusion Assessment Method scale (see below for more details). Patients who did not develop delirium during their postoperative care unit stay or days after surgery were assigned to the ED or POD non-delirium groups, respectively, using propensity score matching with gender, type of surgery, body mass index, Mini-Mental State Examination score, smoking history, drinking history, and preoperative comorbidities (see Figure 1).

We collected their demographic information, smoking and alcohol consumption history, education level, preoperative Mini-Mental State Examination score, complications, and American Society of Anesthesiologists Physical Status Classification, as well as data related to the surgery, such as surgical duration and intraoperative estimated blood loss.

### 2.3. Blood sampling and NfL and GFAP measurements

We obtained blood samples from the patients both before and after their surgery. Prior to surgery, patients rested in a supine position for 10 min after entering the operating room, followed by an arterial puncture and blood sample collection under local anesthesia. Post-surgery, blood samples were collected from the patients 20 min after the endotracheal tube was removed, as well as 24 h after the surgery. The blood samples were collected using EDTA-containing tubes and underwent low-speed centrifugation to separate plasma, which was then aliquoted into frozen storage tubes and stored at −80°C. The samples were processed within 4 h of collection. We used the Simoa HD-x Analyzer (Quanterix, Lexington, MA, USA) to measure the concentrations of NfL and GFAP in the plasma samples, and all measurements were conducted in duplicate. The mean concentration of the two measurements was used for the final data analysis. The intra-assay coefficients of variation for all measurements were < 5%.

### 2.4. Anesthesia and perioperative management

Anesthesia was induced using midazolam, etomidate, propofol, sufentanil, and cisatracurium besylate and then maintained with propofol, remifentanil, cisatracurium besylate, and sevoflurane. The dosage of anesthesia medication used for induction and maintenance during surgery was based on the patient's age and weight. The tidal volume was set at 6–8 ml/kg of body weight, and the end-tidal carbon dioxide partial pressure was maintained between 30 and 35 mmHg by adjusting the respiratory rate. Hemodynamic stability was maintained throughout the surgery, and any reduction or increase in intraoperative blood pressure did not exceed 20% of the baseline. Vasoactive drugs, such as ephedrine and phenylephrine, were used for intervention when necessary to maintain a mean blood pressure of >65 mmHg. The bispectral index was used to monitor the depth of anesthesia, and it was maintained between 40 and 60. Controlled intravenous analgesia with sufentanil and dezocine was used for postoperative pain management.

### 2.5. Assessment of emergence delirium and postoperative delirium

ED was defined as any episodes of delirium during postoperative care unit stay (12). The occurrence of ED was assessed using the Confusion Assessment Method for the Intensive Care Unit primarily at 20 min after tracheal tube removal (27). Prior to the delirium assessment, the Richmond Agitation Sedation Scale (RASS) was used to determine the patient's level of sedation/agitation (28). ED was evaluated if the patient's RASS score reached ≥-3. The Confusion Assessment Method for the Intensive Care Unit assessment included four characteristics: (1) acute changes or fluctuations in the state of consciousness; (2) inattention; (3) altered state of consciousness; and (4) disorganized thinking. Two trained and experienced anesthesiologists performed the assessment. Patients were considered to have delirium if they displayed (1) and (2), plus (3), and/or (4) (29).

Postoperative delirium assessment was conducted twice daily in the morning (8:00–10:00) and afternoon (16:00–18:00) from postoperative day 1 up to postoperative day 7 using the Confusion Assessment Method (30).

### 2.6. Data analysis

The sample size calculation was based on a previous study that demonstrated an increase in postoperative NfL levels compared to preoperative NfL levels (31). Assuming a 33% incidence of delirium, we estimated that 60 patients would be needed to achieve 80% power (α = 5%) to detect a 15 pg/ml difference in NfL levels (standard deviation 24 pg/ml) attributable to delirium. Continuous variables, if normally distributed, were reported as mean ± standard deviation and analyzed using Student's *t*-test. Non-normally distributed continuous variables were reported as median (interquartile range) and compared using the Mann–Whitney *U*-test. The Wilcoxon signed-rank test was used to compare data obtained before and after surgery. Categorical variables were reported as frequencies (%) and compared using chi-square or Fisher's exact tests. Plasma concentrations of NfL and GFAP during surgery were logarithmically transformed with a base of 10 to facilitate statistical inference without altering the relative relationships between the data (32). All preoperative to postoperative change values were calculated as the log10-transformed postoperative value minus the log10-transformed preoperative value (23). Logistic regression analysis was used to adjust for confounding factors and analyze the independent association of plasma biomarkers on delirium. We used SPSS software (version 25.0, IBM, Armonk, New York, USA) for all statistical analyses. All tests were two-sided, and a *P*-value of < 0.05 was considered to be statistically significant.

## 3. Result

At the time of enrollment, 143 patients were excluded for not meeting the inclusion/exclusion criteria. Reasons for exclusion included a diagnosis of cerebral infarction (*n* = 11), Parkinson's disease (*n* = 3), poorly controlled hypertension, diabetes, or coronary heart disease (*n* = 41), severe vision or hearing impairment (*n* = 8), and enrollment in another clinical study (*n* = 29). Additionally, 40 patients declined to participate, and 11 patients were excluded due to other reasons, including surgery cancelation (*n* = 11). Finally, a total of 196 patients were enrolled, of which 134 did not develop delirium, while 62 developed delirium, including 30 with ED and 32 with POD (Figure 1). The total incidence of both types of delirium was 31.63%. The surgical types mainly included cardiac and gastrointestinal surgeries, and the proportion of cardiac and non-cardiac surgeries was consistent between the delirium and non-delirium groups. There was no significant difference in the dosage of various types of anesthetics used in delirious and non-delirious patients during surgery (see Supplementary Table 1). Delirious patients had a lower percentage of class II in the American Society of Anesthesiologists Physical Status Classification and longer surgical duration. The baseline characteristics are summarized in Table 1.

**Table 1:** Adjusted baseline population characteristics of different types of delirium.

**Variable**	**Non-delirium^a^**	**ED**	***P***	**Non-delirium^b^**	**POD**	***P***
	***n* = 30**	***n* = 30**		***n* = 32**	***n* = 32**	
Age (Y)	70.40 ± 4.39	69.03 ± 4.81	0.255	70.31 ± 3.82	69.22 ± 5.00	0.330
Male sex, *n* (%)	17.00 (56.67)	18.00 (60.00)	>0.999	17.00 (53.13)	19.00 (59.38)	0.614
BMI (kg/m^2^)	23.30 ± 2.50	23.09 ± 2.89	0.767	24.16 ± 2.85	22.97 ± 3.72	0.155
Smoke status, *n* (%)	9.00 (30.00)	8.00 (26.67)	>0.999	10.00 (31.25)	9.00 (28.13)	>0.999
Alcohol use, *n* (%)	7.00 (23.33)	6.00 (20.00)	0.701	7.00 (21.88)	5.00 (15.63)	0.351
Education years (Y)	9.00 (6.00–12.00)	8.50 (4.00–12.00)	0.692	9.00 (6.00–12.00)	6.00 (4.00–9.00)	0.236
Preoperative MMSE (score)	25.00 (19.00–27.25)	24.50 (20.75–28.00)	0.578	26.00 (24.00–28.00)	26.00 (24.00–28.00)	0.919
**Preoperative comorbidities**, ***n*** **(%)**						
Hypertension	8.00 (26.67)	9.00 (30.00)	>0.999	16.00 (50.00)	15.00 (46.88)	>0.999
Diabetes	6.00 (20.00)	6.00 (20.00)	>0.999	7.00 (21.88)	3.00 (9.38)	0.302
Coronary heart disease	2.00 (6.67)	2.00 (6.67)	>0.999	7.00 (21.88)	12.00 (37.50)	0.171
**ASA physical status**, ***n*** **(%)**						
II	9.00 (30.00)	2.00 (6.67)	0.023^*^	6.00 (18.75)	1.00 (3.12)	0.175
III	16.00 (53.33)	26.00 (86.66)		16.00 (50.00)	18.00 (56.25)	
IV	5.00 (16.67)	2.00 (6.67)		10.00 (31.25)	13.00 (40.63)	
Surgical duration (min)	199.87 ± 79.83	235.33 ± 68.63	0.070	196.09 ± 94.71	260.16 ± 99.96	0.011^*^
Blood loss (ml)	150.00 (87.50–200.00)	100.00 (100.00–150.00)	0.465	150.00 (50.00–300.00)	250.00 (100.00–400.00)	0.115

Data are presented as n (%), median (IQR), or mean ± SD.

^a^The control group of the ED group obtained from PSM.

^b^The control group of the POD group obtained from PSM.

^*^P < 0.05.

BMI, body mass index; MMSE, Mini-Mental State Examination; ASA, American Society of Anesthesiologists; IQR, interquartile range; SD, standard deviation; PSM, propensity score match.

### 3.1. Preoperative and postoperative NfL and GFAP changes

**Figure 2:**
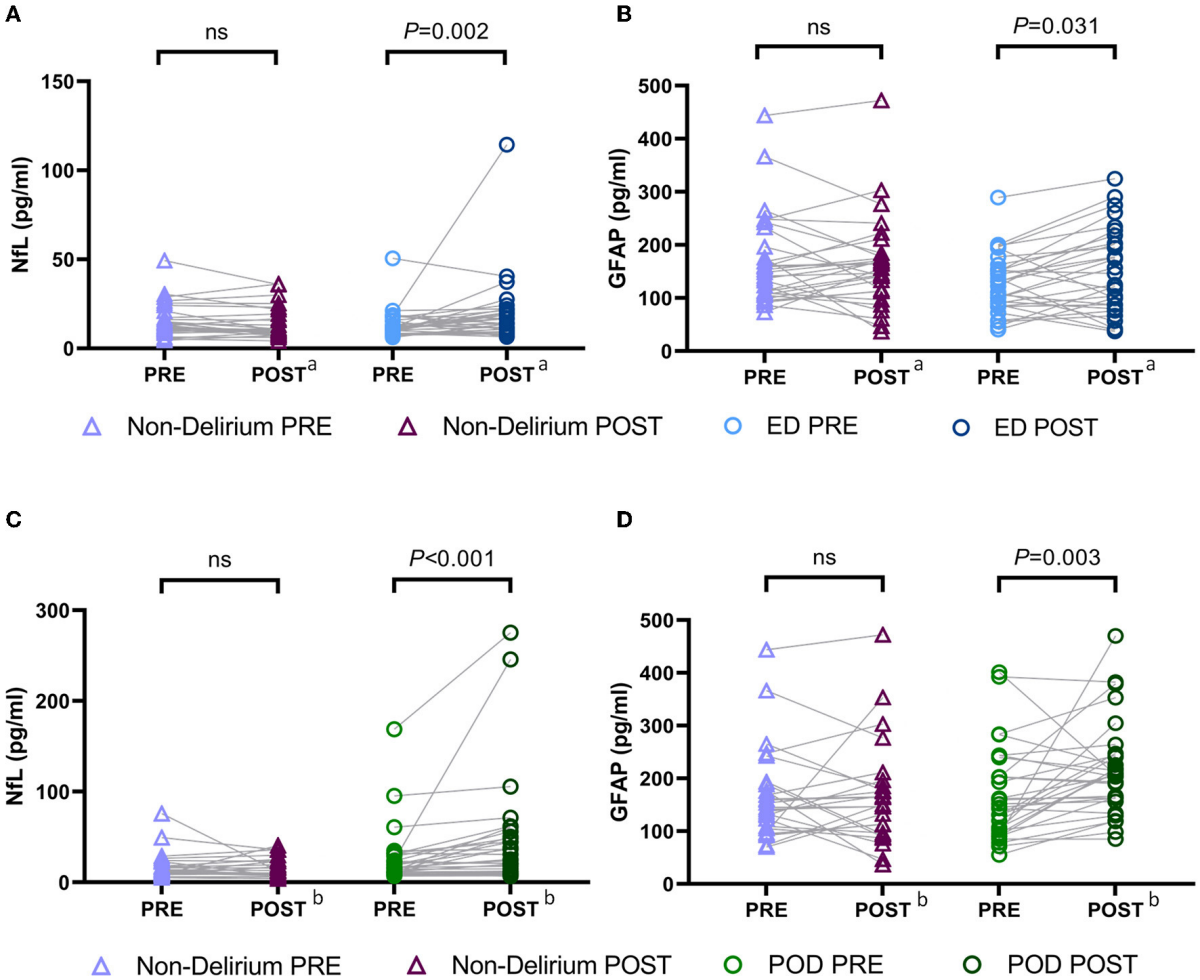
Preoperative and postoperative NfL and GFAP levels in patients with and without delirium. **(A)** Preoperative and postoperative NfL levels in patients with emergence delirium and patients without delirium; **(B)** preoperative and postoperative GFAP levels in patients with emergence delirium and patients without delirium; **(C)** preoperative and postoperative NfL levels in patients with postoperative delirium and patients without delirium; **(D)** preoperative and postoperative GFAP levels in patients with postoperative delirium and patients without delirium. ^a^Blood samples obtained immediately after surgery; ^b^blood samples obtained on the postoperative day 1. NfL, neurofilament light chain; GFAP, glial fibrillary acidic protein; PRE, preoperative; POST, postoperative; ED, emergence delirium; POD, postoperative delirium.

Before the surgery, we measured the concentrations of NfL and GFAP in the plasma of all patients and found no significant difference in the preoperative measurements between patients with and without delirium (Figure 2).

Following surgery, there were significant increases in the concentrations of plasma NfL and GFAP in both ED and POD patients. Notably, in the ED group, compared to preoperative plasma NfL and GFAP levels, postoperative concentrations of NfL [preoperative vs. postoperative: 11.57 (9.99–16.11) pg/ml vs. 15.50 (10.72–21.50) pg/ml, *P* = 0.022] and GFAP [preoperative vs. postoperative: 126.3 (86.74–156.80) pg/ml vs. 135.80 (85.03–207.00) pg/ml, *P* = 0.031] were significantly elevated (Figures 2A, B). Similarly, in POD patients, postoperative concentrations of plasma NfL [preoperative vs. postoperative: 16.17 (10.78–27.83) pg/ml vs. 24.75 (10.93–49.97) pg/ml, *P* < 0.001] and GFAP [preoperative vs. postoperative: 145.20 (95.38–203.00) pg/ml vs. 207.00 (162.60–243.30) pg/ml, *P* = 0.003] were also significantly increased compared to preoperative levels (Figures 2C, D). However, we did not observe significant changes in plasma NfL and GFAP concentrations during general anesthesia surgery in patients without delirium.

**Figure 3:**
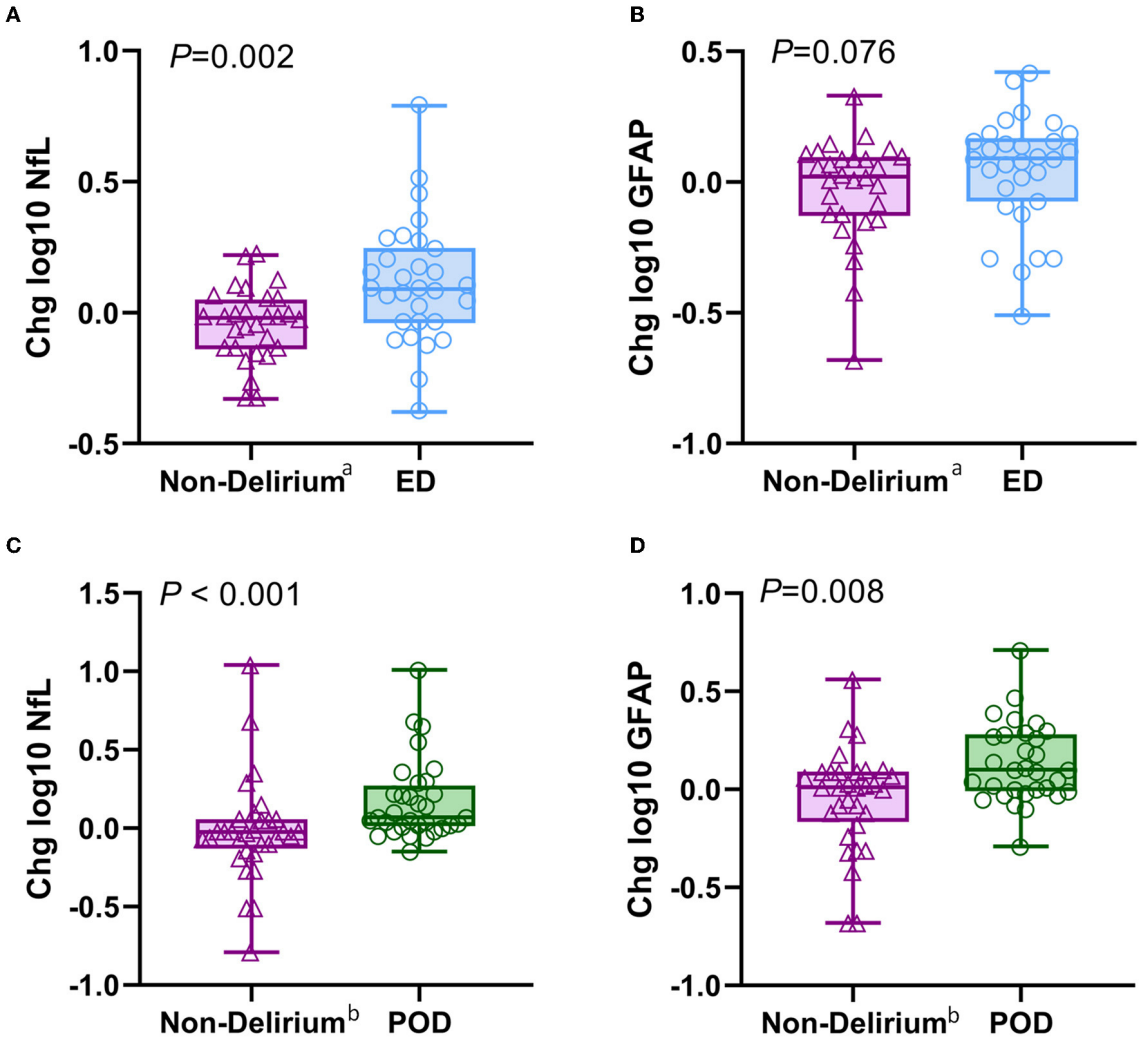
Changes of intraoperative NfL and GFAP levels in patients with different types of delirium. **(A, B)** Intraoperative changes in plasma NfL and GFAP between the ED and non-delirium patients (*n* = 60). **(C, D)** Intraoperative changes in plasma NfL and GFAP between the POD and non-delirium patients (*n* = 64). The changes were normalized by log10-transforming the postoperative value and the baseline value and then subtracting the transformed preoperative value from the transformed postoperative value. ^a^The control group of the ED group obtained from PSM; ^b^the control group of the POD group obtained from PSM. NfL, neurofilament light chain; GFAP, glial fibrillary acidic protein; ED, emergence delirium; POD, postoperative delirium.

Furthermore, we compared the intraoperative changes in plasma concentrations of NfL and GFAP between delirious and non-delirious patients. As described in Section 2.6 of the Methods, all changes were logarithmically transformed (base 10). We found that the increase in NfL levels in ED patients was significantly higher than in non-delirium patients [non-delirium vs. ED: −0.02 (−0.14–0.05) vs. 0.09 (−0.04–0.25), *P* = 0.002, Figure 3A], whereas the change in GFAP levels did not differ significantly between the two groups (*P* = 0.076, Figure 3B). In POD patients, the intraoperative increases in both plasma NfL [non-delirium vs. POD: −0.03 (−0.13–0.06) vs. 0.07 (0.01–0.29), *P* < 0.001] and GFAP [non-delirium vs. POD: 0.01 (−0.16–0.09) vs. 0.10 (−0.01–0.28), *P* = 0.008] were significantly higher than in non-delirium patients (Figures 3C, D).

**Figure 4:**
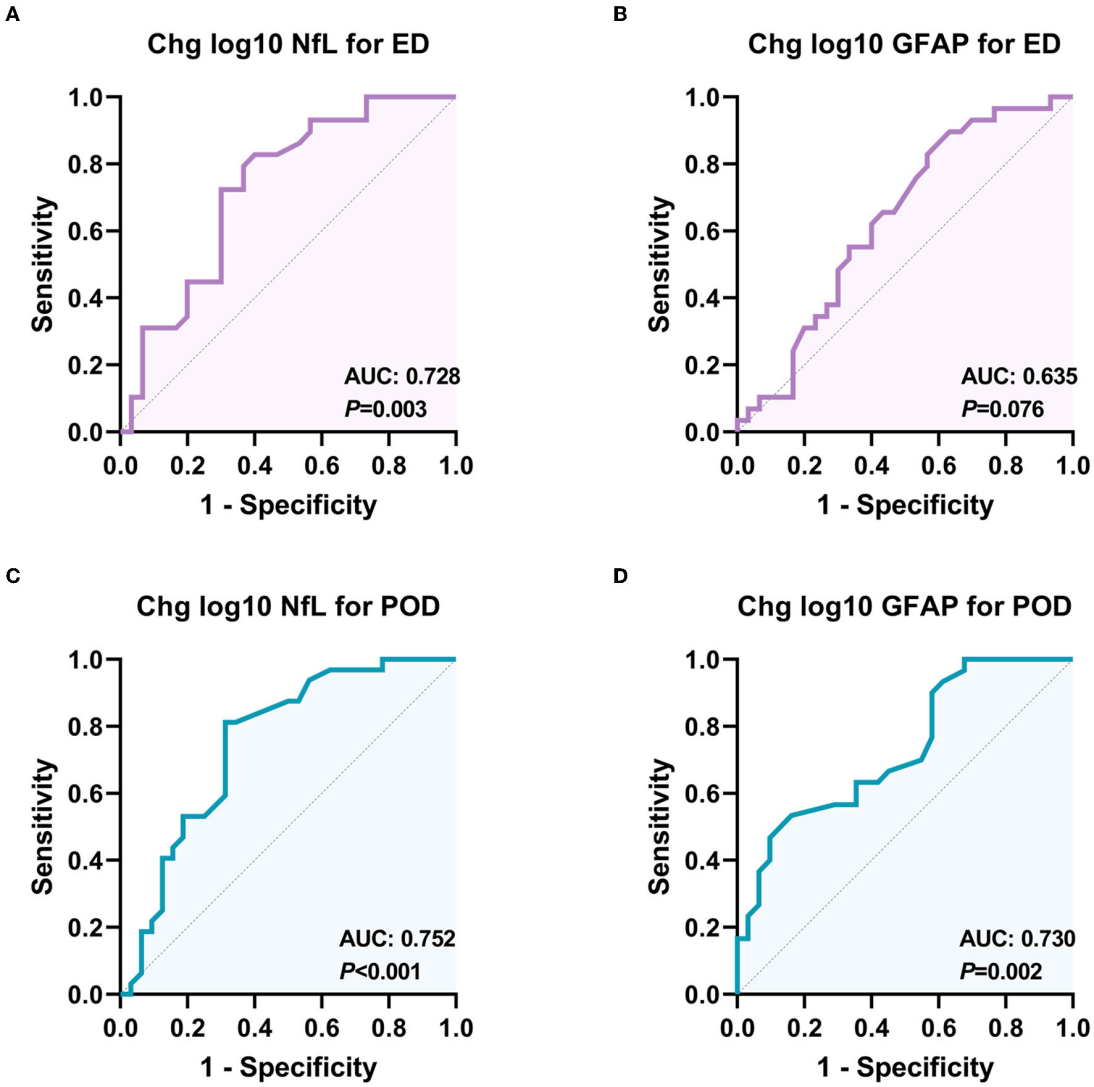
Receiver operating characteristic (ROC) curves for the diagnostic value of NfL and GFAP changes for different delirious types. **(A)** ROC curve for the classification ability of plasma NfL to distinguish ED from non-delirium patients (*n* = 60); **(B)** ROC curve for the classification ability of plasma GFAP to distinguish ED from non-delirium patients (*n* = 60); **(C)** ROC curve for the classification ability of plasma NfL to distinguish PD from non-delirium patients (*n* = 64); **(D)** ROC curve for the classification ability of plasma GFAP to distinguish PD from non-delirium (*n* = 64). ED, emergence delirium; PD, postoperative delirium; NfL, neurofilament light chain; GFAP, glial fibrillary acidic protein.

As shown in Figure 4, the analysis of the receiver operating characteristic curve results showed that the intraoperative change in plasma NfL, rather than GFAP, has a high predictive value for ED. In addition, the intraoperative changes in plasma NfL and GFAP both have predictive values for the occurrence of POD.

### 3.2. Association of NfL and GFAP changes with different types of delirium

Univariate analysis for ED and POD is presented in Supplementary Table 2. The results of multivariable logistic regression analysis revealed that ASA status = III (adjusted OR = 11.577, *P* = 0.009) and a higher NfL change (adjusted OR = 1.872, *P* = 0.005) were significantly associated with ED (as shown in Table 2). However, the GFAP change did not show a significant association with ED. Although patients who developed POD had a more significant increase in postoperative NfL and GFAP levels, the multivariable logistic regression analysis only showed that the change in GFAP, not NfL during surgery, was independently associated with the development of POD (adjusted OR = 1.419, *P* = 0.016, as shown in Table 2). Moreover, longer surgical duration was also associated with POD (adjusted OR = 1.006, *P* = 0.045).

**Table 2:** Multivariable adjusted risk factors for emergence delirium (*n* = 60) and postoperative delirium (*n* = 64).

**Variable**		**β**	***P***	**a OR**
ED	Plasma NfL^a^	0.627	0.005^**^	1.872
	**ASA physical status**			
	II	Reference	Reference	Reference
	III	2.449	0.009^**^	11.577
	IV	1.407	0.255	4.085
POD	Plasma GFAP^b^	0.350	0.016^*^	1.419
	Surgical duration	0.006	0.045^*^	1.006

NfL and GFAP were normalized by log_10_-transforming the postoperative value and the baseline value and then subtracting the transformed preoperative value from the transformed postoperative value.

^a^For every 0.1-unit increase in the logarithmic transformation value of the change in NfL during the surgery.

^b^For every 0.1-unit increase in the logarithmic transformation value of the change in GFAP during the surgery.

^*^P < 0.05.

^**^P < 0.01.

β, coefficient; a OR, adjusted odds ratio; NfL, neurofilament light chain; GFAP, glial fibrillary acidic protein; ASA, American Society of Anesthesiologists.

## 4. Discussion

An abundance of research studies has shown that blood biomarkers, such as NfL and GFAP, are promising indicators for monitoring central nervous system diseases. In our study, we compared changes in plasma NfL and GFAP concentrations during surgery between delirious and non-delirious patients. We found a significant increase in NfL in ED patients, and both plasma NfL and GFAP levels increased in POD patients. Our data suggest that emergence delirium is associated with a more significant axonal injury during surgery, while postoperative delirium is accompanied by activation of the astrocyte.

It is widely believed that general anesthesia and surgery may cause brain dysfunction in older patients (33). During surgical procedures, activation of systemic inflammation (34), inadequate organ perfusion, hypotension (35), and other factors can cause intraoperative brain injury. Furthermore, as general anesthetics affect receptors, such as GABAA and NMDA receptors, which are widely present in mammalian brains, the off-target effects of these anesthetics can lead to adverse outcomes related to cognitive impairment (36). At older ages, the reduced compensating capacity of aging brains may make them more susceptible to the unwanted effects of general anesthetics. Due to the invasiveness of cerebrospinal fluid sampling and the high cost of imaging tests, detecting blood biomarkers provides a more convenient way to quantify brain injury during general anesthesia in clinical practice.

Emergence delirium is a type of acute cognitive dysfunction that occurs during the early period of recovery after undergoing general anesthesia (9). To the best of our knowledge, no studies have investigated the correlation between emergence delirium and NfL and GFAP levels during general anesthesia. Our study did not observe differences in preoperative plasma NfL levels between the ED and non-delirium groups. However, after surgery, NfL levels in the ED group were significantly increased compared to the non-delirium group. Normally, a small amount of NfL is slowly released from axons into the cerebrospinal fluid. However, in cases of axonal injury due to inflammation, trauma, vascular injury, and others, the release of NfL rapidly increases and enters the peripheral bloodstream through the exchange between cerebrospinal fluid and blood (16). Imaging results suggest that axonal injury may be one of the pathological mechanisms of cognitive impairment (37). Animal experiments have also shown that improving axonal injury can improve the cognitive function of rats with traumatic brain injury (38). Our data support the association between neuronal axonal injury and cognitive dysfunction. The occurrence of neuronal axonal injury may also be one of the reasons for the long-term cognitive decline in delirium patients (39).

Postoperative delirium is a type of acute cognitive dysfunction that typically manifests within 24–72 h following surgery. In our study, we found that the changes in NfL and GFAP levels on the 1st day after surgery were still higher in patients with POD than in non-delirium patients, supporting our hypothesis that neuronal axonal injury plays a role in the development of delirium. Our data also suggest that astrocyte activation may be involved in the occurrence of acute delirium. Astrocytes participate in many important central nervous system functions, including neuronal survival and differentiation, energy metabolism, and immune defense (40, 41). The activation of astrocytes is accompanied by an increase in GFAP levels, and this activation has been implicated in neuroinflammation (42, 43). Some studies have demonstrated that astrocytes can act as a strong source of complement components, cytokines, such as IL-1β, IL-6, and chemokines, including CCL2, CXCL1, CXCL10, and CXCL12, in response to various stimuli (44). While previous studies have reported conflicting findings regarding the changes in GFAP levels during surgery and postoperative delirium (24, 45), most studies suggest that neuroinflammation associated with GFAP activation plays a key role in the development of delirium (46). However, these discrepancies may be due to differences in study design, surgical types, and patient populations, and further research is necessary to clarify this issue.

NfL has been shown to be useful for diagnosing, monitoring, and predicting various neurological disorders (26). Several previous studies have reported an association between NfL and delirium. Research on hip fracture surgery found significantly elevated serum NfL levels in delirium patients both preoperatively and postoperatively compared to non-delirium patients (47), and preoperative NfL levels were associated with neurodegeneration detected by brain MRI (21). Although we did not observe any significant differences in preoperative plasma NfL levels between delirium and non-delirium patients in our study, we cannot rule out the potential link between preoperative NfL levels and delirium. Furthermore, larger studies are needed to confirm any potential associations. Of particular note, this study is, to the best of our knowledge, the first to investigate the relationship between emergence delirium and plasma NfL levels. Our findings provide initial evidence for an association between emergence delirium and axonal injury. Furthermore, there is no current evidence supporting a link between preoperative GFAP levels and postoperative delirium. Our study observed a significant increase in GFAP levels in delirium patients, suggesting the involvement of astrocyte activation in delirium development, which could be a new target for postoperative delirium therapy.

In this study, we employed the Simoa technology as an alternative to traditional enzyme-linked immunosorbent assay and electrochemiluminescence for detecting protein biomarkers in blood at sub-femtomolar concentrations (16, 48, 49). Simoa technology is ultra-sensitive, allowing for more precise detection of biomarkers and showing promise for early disease monitoring and follow-up. However, while biomarkers' sensitive characteristics aid in early disease diagnosis (27), their potential value lies not in distinguishing between postoperative neurological complications that have a similar axonal injury or astrocyte activation but in assessing the severity of the injury. Therefore, clinical diagnoses of delirium should be complemented with assessments of other neurological functions or imaging results.

This study has several limitations. First, the sample size was relatively small, and a larger sample size is needed for validation. Second, although we attempted to control some confounding factors, we only controlled a limited number of variables. For example, during the case collection stage, we controlled patient age and surgical type; during propensity score matching, we controlled patient gender, BMI, preoperative cognitive score, smoking and alcohol history, and preoperative complications; finally, during the multivariate analysis stage, we controlled patients' ASA classification and surgical duration. However, there may still be some unmeasured confounding that is a problem for any observational study. Based on this study, we have discovered a preliminary association between the occurrence of delirium and perioperative neural axonal injury and activation of astrocytes. In subsequent studies, we intend to establish a larger prospective cohort to identify possible correlations between pre- and postoperative blood biomarkers and the incidence of delirium and long-term cognitive impairment to the greatest extent possible. However, it is important to note that observational studies cannot establish causality. Further research, such as interventional experiments to investigate whether treatments targeting axonal injury and activation of astrocytes will benefit elderly patients after surgery, is also necessary.

## 5. Conclusion

In summary, our findings suggest that emergence delirium is linked to elevated levels of NfL, a biomarker of axonal injury, during surgery. Additionally, the rise in GFAP, a marker of astrocyte activation, is also associated with postoperative delirium. We recommend further validation of these results in a larger sample size and investigation of their potential long-term cognitive implications.

## Data availability statement

The raw data supporting the conclusions of this article will be made available by the authors, without undue reservation.

## Ethics statement

The studies involving human participants were reviewed and approved by Ethics Committee of Xiangya Hospital Central South University (201907296). The patients/participants provided their written informed consent to participate in this study.

## Author contributions

EW: study design. XL, YW, and CY: patient recruitment and data collection. XL and YW: drafting of the manuscript. JW: statistical analysis. DM and EW: critical revision. All authors contributed to the article and approved the submitted version.
