# Supplementary material for: Emergence delirium and postoperative delirium associated with high plasma NfL and GFAP: an observational study
Source: Front Med (Lausanne). 2023 Jul 28;10:1107369. doi: 10.3389/fmed.2023.1107369 (PMC10419211; doi:10.3389/fmed.2023.1107369)
Supplement: Supplementary file 1 [file Table_1.docx]

**Supplemental Table 1 The anesthetic drugs usages of different types of delirium.**

| **Variable** | **Non-**  **delirium ^a^**  **n=30** | **ED**  **n=30** | ***P*** | **Non-**  **delirium ^b^**  **n=32** | **POD**  **n=32** | ***P*** |
| --- | --- | --- | --- | --- | --- | --- |
| **Midazolam** | 2.00  (2.00-3.00) | 2.00  (1.00-3.00) | 0.500 | 2.50  (2.00-3.00) | 2.00  (2.00-3.00) | 0.211 |
| **Etomidate** | 20.00  (16.00-20.00) | 20.00  (15.00-20.00) | 0.396 | 20.00  (16.00-20.00) | 20.00  (16.00-20.00) | 0.938 |
| **Propofol** | 800.00  (500.00-1000.00) | 800.00  (635.00-1200.00) | 0.306 | 600.00  (300.00-800.00) | 600.00  (500.00-800.00) | 0.509 |
| **Sufentanil** | 50.00  (37.50-60.00) | 50.00  (50.00-62.50) | 0.295 | 40.00  (30.00-50.00) | 50.00  (50.00-50.00) | 0.223 |
| **Remifentanil** | 1.00  (0.50-2.00) | 1.50  (1.00-2.00) | 0.147 | 0.95  (0.50-1.00) | 1.00  (1.00-2.00) | 0.141 |

Data are presented as median (IQR). ^a^ the control group of the ED group obtained from PSM; ^b^ the control group of the POD group obtained from PSM.
