# Supplementary material for: Emergence delirium and postoperative delirium associated with high plasma NfL and GFAP: an observational study
Source: Front Med (Lausanne). 2023 Jul 28;10:1107369. doi: 10.3389/fmed.2023.1107369 (PMC10419211; doi:10.3389/fmed.2023.1107369)
Supplement: Supplementary file 2 [file Table_2.docx]

**Supplemental Table 2 Unadjusted analysis of risk factors for emergence delirium (n = 60) and postoperative delirium (n = 64).**

| **Variable** | | **β** | ***P*** | **OR** |
| --- | --- | --- | --- | --- |
| **ED** | **NfL change log10 ^a^** | 0.497 | 0.007** | 1.644 |
|  | **GFAP change log10 ^b^** | 0.177 | 0.185 | 1.193 |
|  | **ASA physical status** | | | |
|  | **Ⅱ** | Reference | Reference | Reference |
|  | **Ⅲ** | 1.990 | 0.018* | 7.312 |
|  | **Ⅳ** | 0.588 | 0.608 | 1.800 |
|  | **Surgical duration** | 0.007 | 0.074 | 1.007 |
| **POD** | **NfL change log10 ^a^** | 0.268 | 0.021* | 1.308 |
|  | **GFAP change log10 ^b^** | 0.381 | 0.009** | 1.463 |
|  | **Surgical duration** | 0.007 | 0.015* | 1.007 |

NfL and GFAP were normalized by log10-transforming the postoperative value and the baseline value and then subtracting the transformed preoperative value from the transformed postoperative value. ^a^ For every 0.1-unit increase in the logarithmic transformation value of the change in NfL during the surgery; ^b^ For every 0.1-unit increase in the logarithmic transformation value of the change in GFAP during the surgery. **P* < 0.05, ** *P* < 0. 01. Abbreviation: β, coefficient; OR, Odds Ratio; NfL, Neurofilament light chain; GFAP, Glial fibrillary acidic protein; ASA, American Society of Anesthesiologists.
